# Cellular analysis of cleavage-stage chick embryos reveals hidden conservation in vertebrate early development

**DOI:** 10.1242/dev.118604

**Published:** 2015-04-01

**Authors:** Hiroki Nagai, Maiko Sezaki, Kisa Kakiguchi, Yukiko Nakaya, Hyung Chul Lee, Raj Ladher, Tomohiro Sasanami, Jae Yong Han, Shigenobu Yonemura, Guojun Sheng

**Affiliations:** 1 Laboratory for Early Embryogenesis, RIKEN Center for Developmental Biology, Kobe, Hyogo 650-0047, Japan; 2 Electron Microscopy Laboratory, RIKEN Center for Developmental Biology, Kobe, Hyogo 650-0047, Japan; 3 Department of Agricultural Biotechnology, Seoul National University, Seoul 151-921, Korea; 4 Laboratory for Sensory Development, RIKEN Center for Developmental Biology, Kobe, Hyogo 650-0047, Japan; 5 Department of Applied Biological Chemistry, Faculty of Agriculture, Shizuoka University, 836 Ohya, Shizuoka 422-8529, Japan

**Keywords:** Amniote, Cellularization, Chick, Cleavage, Yolk syncytium, Zygotic gene activation

## Abstract

Birds and mammals, phylogenetically close amniotes with similar post-gastrula development, exhibit little conservation in their post-fertilization cleavage patterns. Data from the mouse suggest that cellular morphogenesis and molecular signaling at the cleavage stage play important roles in lineage specification at later (blastula and gastrula) stages. Very little is known, however, about cleavage-stage chick embryos, owing to their poor accessibility. This period of chick development takes place before egg-laying and encompasses several fundamental processes of avian embryology, including zygotic gene activation (ZGA) and blastoderm cell-layer increase. We have carried out morphological and cellular analyses of cleavage-stage chick embryos covering the first half of pre-ovipositional development, from Eyal-Giladi and Kochav stage (EGK-) I to EGK-V. Scanning electron microscopy revealed remarkable subcellular details of blastomere cellularization and subgerminal cavity formation. Phosphorylated RNA polymerase II immunostaining showed that ZGA in the chick starts at early EGK-III during the 7th to 8th nuclear division cycle, comparable with the time reported for other yolk-rich vertebrates (e.g. zebrafish and *Xenopus*). The increase in the number of cell layers after EGK-III is not a direct consequence of oriented cell division. Finally, we present evidence that, as in the zebrafish embryo, a yolk syncytial layer is formed in the avian embryo after EGK-V. Our data suggest that several fundamental features of cleavage-stage development in birds resemble those in yolk-rich anamniote species, revealing conservation in vertebrate early development. Whether this conservation lends morphogenetic support to the anamniote-to-amniote transition in evolution or reflects developmental plasticity in convergent evolution awaits further investigation.

## Abstract (precis)

**Summary:** Early chick embryos share previously unappreciated features with anamniote embryos such as the timing of zygotic gene activation and yolk syncytial layer formation.

## INTRODUCTION

A mature avian oocyte is fertilized in the infundibulum shortly after ovulation. From fertilization to egg-laying, the period of pre-ovipositional development covers ∼25 h in the chick, during which a number of important early embryological events take place. These events include the meroblastic cleavage, cellularization, zygotic gene activation, blastoderm layer increase and reduction, radial symmetry breaking, and segregation of epiblast and hypoblast lineages (Sheng, 2014). Owing to poor accessibility, no comprehensive molecular or cellular study on pre-ovipositional embryos has been reported in the literature. General morphological characterization in the 1970s by Eyal-Giladi and Kochav provided us with a basic staging system, the EGK stages (Eyal-Giladi, 1991; Eyal-Giladi and Kochav, 1976; Kochav et al., 1980), and the ultrastructure of embryos from the later half of this developmental period was investigated in the 1990s with electron microscopy by Etches and colleagues (Watt et al., 1993).

According to the EGK staging system, pre-streak chick development is divided into 14 sub-stages, from EGK-I to EGK-XIV. The pre-ovipositional period includes the stages EGK-I to EGK-X. These 10 stages are further grouped into two developmental phases. In the first, from EGK-I to EGK-VI (the cleavage stages), a chick embryo grows mainly by means of cell proliferation (1→10,000) and blastoderm cell-layer increase (1→6). In the second, from EGK-VII to EGK-X (the area pellucida formation stages), the center of the blastoderm above the subgerminal cavity thins out from six to two cell layers, leading to the formation of two circular territories: the central area pellucida, which is detached from the yolk and contains the epiblast and hypoblast cell layers; and the peripheral area opaca, which varies in its thickness and maintains cellular interactions with the yolk. Radial symmetry breaking, one of the most important developmental events in vertebrate embryogenesis, also occurs during this phase.

With a paucity of molecular and cellular understanding, and with the exception of primordial germ cells (PGCs) (Tsunekawa et al., 2000), chick embryos during the first phase of pre-ovipositional development are generally regarded as being undifferentiated, containing equipotent cells whose morphological variations reflect their cellularization and proliferation heterogeneity rather than their fate diversification. These embryos are also considered to be inactive in their preparation for lineage segregation (except for the PGCs) or radial symmetry breaking because embryonic axis can be efficiently re-specified with physical perturbation during the second phase of pre-ovipositional development (Kochav and Eyal-Giladi, 1971). Data from other vertebrate models, however, suggest that molecular and cellular events taking place during cleavage phase of the early development are crucial for dynamic signaling, lineage specification and morphogenetic movements associated with the late blastula/early gastrula development.

Among those crucial events are the initiation of zygotic gene activation (ZGA), the separation of outside and inside blastomeres, and the formation of a yolk syncytial layer (YSL). In both mouse and zebrafish embryos, ZGA comes under direct control of key pluripotency regulators (Foygel et al., 2008; Lee et al., 2013b; Leichsenring et al., 2013) and is tightly associated with epigenetic reprogramming of parental genomes that, later in development, controls the specification of both somatic and germ cell lineages (Hackett and Surani, 2013). During mouse early development, 8→16 and 16→32 divisions produce two types of blastomeres: those located outside, which are biased towards the trophectoderm fate; and those located inside, which will become the inner cell mass (Rossant and Tam, 2009). This process is regulated by polarized cell division and post-divisional cell rearrangement (Johnson and Ziomek, 1981; Parfitt and Zernicka-Goetz, 2010; Sasaki, 2010; Watanabe et al., 2014). The YSL is a special adaptive feature of telolecithal development. Although eutherian eggs are devoid of yolk, both birds and reptiles, two other extant amniote groups, have telolecithal eggs. Studies in zebrafish, a genetic model for telolecithal embryonic development, suggest that the YSL is formed at the cleavage stage and that it undergoes highly coordinated cytokinetic movements with the overlying blastoderm cells (Carvalho and Heisenberg, 2010). It also regulates mesendoderm induction by secreting Ndr1 and Ndr2 signals (two nodal related molecules) under the transcriptional control of Mxtx2 (Hong et al., 2011), and produces the ventralizing signal BMP2b under the transcriptional control of MGA, MAX and Smad4 proteins (Sun et al., 2014). To investigate how these events occur in the chick and whether novel insights into vertebrate early development can be gained by taking a comparative approach, we collected cleavage-stage chick embryos and performed in-depth morphological and cell biological analyses.

## RESULTS AND DISCUSSION

### General description of EGK-I to -V embryos

Pre-ovipositional eggs were retrieved from laying hens using a non-invasive method described previously (Eyal-Giladi and Kochav, 1976; Lee et al., 2013a). Because the ultrastructure of late cleavage-stage embryos and the area pellucida of formation-stage embryos had been described previously (Watt et al., 1993), we focused our attention on the early and mid-cleavage stages, namely from EGK-I to EGK-V. This period covers about 8 hours of development. Together with time needed from the fertilization to the first cell division, it is considered here to represent the first half of pre-ovipositional development (∼13 h out of a total of 25 h). After egg retrieval, shell and albumen were removed, and embryos were briefly fixed in 4% PFA with intact yolk for morphological preservation. They were then cut out together with a small amount of underlying yolk to retain embryo-yolk contacts, further fixed in 4% PFA and stored at 4°C for later analysis. Out of a total of 138 embryos collected, 73 were confirmed under a stereo-microscope to represent stages between EGK-I and EGK-V (supplementary material Fig. S1). Their general morphology is briefly described here. At EGK-I, all cells are connected to the yolk basally; when viewed from the apical side, they are not yet enclosed by lateral cell membrane, except for a few centrally located ones at late EGK-I. At EGK-II, two types of cells, the central ones with closed lateral membrane and peripheral ones which are open to the yolk laterally, can be easily distinguished. From EGK-III to EGK-V, the central, closed cells become progressively smaller as they divide, and the area they occupy expands as more closed cells are produced from the peripheral open cells after division. In a majority of embryos at all EGK stages, the central cell cluster is positioned eccentrically in the blastoderm. Compared with those described by Eyal-Giladi and Kochav (1976), our embryos had their morphology better preserved and, likely as a result of this, we did not notice any obvious shrinkage in blastoderm size at EGK-III or large vacuoles at EGK-I and EGK-II, as described previously (Eyal-Giladi and Kochav,1976).

### Scanning electron microscopy of EGK-I to -V embryos

**Fig. 1.**
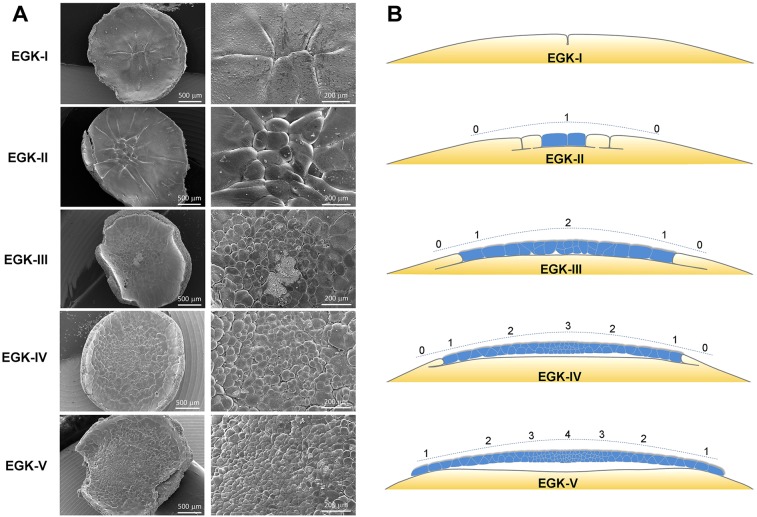
**Scanning electron microscopy analysis of EGK-I to -V chick embryos.** (A) Apical views of EGK-I to -V embryos under SEM. Scale bars: 500 µm (left); 200 µm (right). Details of the fracture surface are shown in supplementary material Figs S2-S6 and summarized in B. (B) Schematic view of cellularization and cell proliferation processes during chick development from EGK-I to -V. Completely cellularized blastomeres are shown in blue. Numbers above the blastoderm indicate the distribution of cell layer numbers.

To gain a better understanding of cellular morphogenesis during early cleavages, we performed scanning electron microscopy (SEM) analysis with EGK-I to EGK-V embryos (Fig. 1A; supplementary material Figs S2-S6; summarized in Fig. 1B). Under SEM, cleavage furrows and cell boundaries were easily visible. In an EGK-I embryo with eight open cells (Fig. 1A; supplementary material Fig. S2), the order of three separate cytokinetic events could be estimated based on the shape and extent of furrow progression. Fracture surface views indicated that transition from vertical furrow burrowing to horizontal furrow burrowing took place ∼50-100 µm deep into the yolk (supplementary material Fig. S2C-E). The transition area had elaborate membrane protrusions (supplementary material Fig. S2E), resembling the furrow based body (Bellairs et al., 1978; Gipson, 1974). The apical surface was rich in microvilli, with small vesicular bodies at the cortex (supplementary material Fig. S2F). Yolk granules exhibited fine-graded distribution in their sizes. No obvious transition zone could be distinguished between yolk granules to be included in future blastoderm cells (<100 µm below the membrane) and those to be allocated to the yolk (supplementary material Fig. S2D,G). In an EGK-II embryo with 16 laterally closed cells (Fig. 1A; supplementary material Fig. S3), the center of closed cells was located clearly off the geometric center of the blastoderm, possibly as a consequence of eccentric localization of the zygotic nucleus. It is unclear whether this eccentricity has any predictive value with regard to where the future posterior side of the epiblast (dorsal side of the embryo) will be. The shape and size of central cells was variable (supplementary material Fig. S3B). Contrary to what had been suggested based on light microscopy analysis (Eyal-Giladi and Kochav, 1976), a few central cells at EGK-II had already finished the cellularization process with complete basal and lateral membranes (supplementary material Fig. S3C,D). Numerous small, intracellular vesicular bodies were present in all cells (supplementary material Fig. S3D,E), resembling those reported in other yolk-rich vertebrate embryos (Danilchik et al., 2003; Li et al., 2006). These vesicles are likely to be involved in the cellularization and cell division processes, which require a constant supply of new plasma membrane. It is also possible, however, that they are related to the supply of surface proteins or to the secretion of extracellular matrix molecules.

At EGK-III (Fig. 1A; supplementary material Fig. S4), the territory of laterally closed cells from apical surface view expanded significantly as more peripheral open cells gave rise to laterally closed daughter cells after division (supplementary material Fig. S4A-C). The apical membrane remained rich in microvilli (supplementary material Fig. S4D), but with reduced abundance at cleavage furrows. A small subgerminal cavity space appeared under some central cells (supplementary material Fig. S4E) and two-cell thick blastoderm was observed occasionally (supplementary material Fig. S4F). This trend continued at EGK-IV (Fig. 1A; supplementary material Fig. S5). At this stage, the subgerminal cavity space became obvious under most central cells and started to merge to form a bona fide cavity above the yolk cell surface (supplementary material Fig. S5C-E). A significant proportion of the blastoderm became two cells thick (supplementary material Fig. S5C,D), with the very central part being three cells thick (supplementary material Fig. S5E). In slightly more peripheral regions, the subgerminal cavity under newly closed cells had just appeared as an isolated pocket between two closed cells and the yolk cell (supplementary material Fig. S5H). At the basal surface of these cells, two closely apposed plasma membranes (supplementary material Fig. S5H′, arrows) marked the completion of their cellularization. Interestingly, unlike the fine-graded size distribution before cellularization (supplementary material Fig. S2G), yolk granules at the basal cortex of newly formed cells were much smaller than those located in the adjacent yolk cells (supplementary material Fig. S5H′, arrowheads). At EGK-V (Fig. 1A; supplementary material Fig. S6), all non-edge cells of the blastoderm had completed the cellularization process. Many edge cells had also become fully cellularized (supplementary material Fig. S6C,E), although some of them remained open peripherally. A smooth transition in blastoderm thickness, from one cell layer at the edge to four cell layers at the center, could be observed (supplementary material Fig. S6C,D). Cells bridging the one-cell and two-cell regions often exhibited wedge-shaped morphology (supplementary material Fig. S6C,F). The subgerminal cavity became a continuous, expanded space below the multilayered part of the blastoderm, but the one-cell layered part remained in close association with the underlying yolk cell membrane (supplementary material Fig. S6C-E). Cell-cell contacts among blastomeres were prominent in both superficial and deep layers (supplementary material Fig. S6G-J).

### Zygotic gene activation (ZGA) is initiated at late EGK-II/early EGK-III

**Fig. 2.**
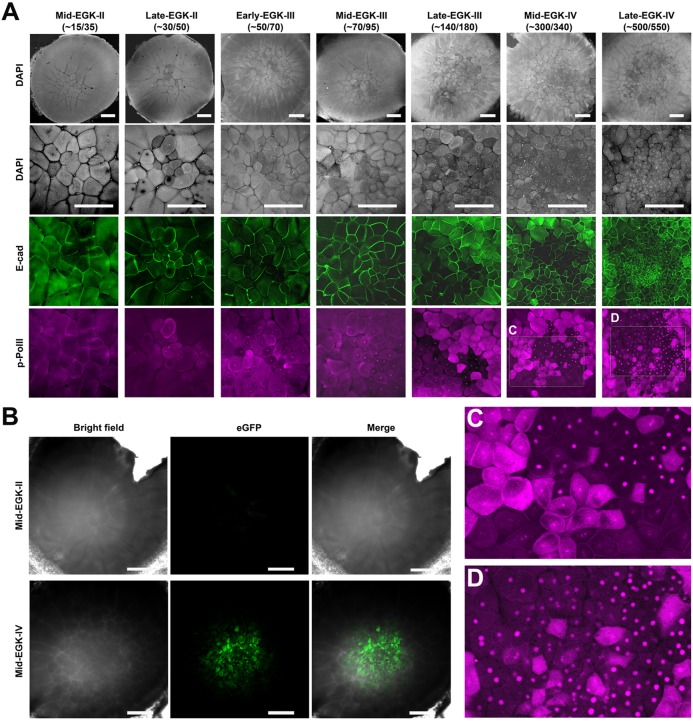
**ZGA in EGK-II to -IV chick embryos analyzed by anti-Ser5 phosphorylation of Pol II CTD (p-PolII) antibody staining and by GFP transgenesis.** (A) Wild-type embryos ranging from mid-EGK-II to late EGK-IV are stained for p-PolII signals. Embryos are co-stained for E-cadherin (E-cad; marking cell boundaries) and DAPI (marking nuclei). Numbers (x/y) under embryo stage labels indicate approximate counts of laterally closed cells (x) and total cells (y). (Top) Whole-embryo views after DAPI staining. At higher magnification: DAPI (second row), E-cad (third row) and p-PolII (bottom row) staining. Signals for p-PolII are negative at mid-EGK-II, very weak in late EGK-II, rapidly increase at EGK-III and become very strong at late EGK-IV. From late EGK-III to late EGK-IV, strong signals are detected both in the nucleus (in some central blastomeres) and in the cytoplasm (in adjacent blastomeres), although the proportion of cytoplasmic-positive cells decreases gradually (C,D). p-PolII-negative, small blastomeres are also observed in central areas next to similarly sized positive blastomeres (C). Signals in peripherally positioned blastomeres are much weaker or negative. (B) Embryos from GFP-transgenic male and wild-type female crosses show strong GFP signals at mid-EGK-IV but no GFP signals at mid-EGK-II. Scale bars: 500 µm.

The cellular morphogenetic process from EGK-I to EGK-V is summarized in Fig. 1B. During this period of chick development, cell (nucleus) number increases from 1 to ∼2000 (Park et al., 2006). ZGA in mammalian embryos (e.g. in the mouse and human) starts very early, before the 3rd cell cycle (Vassena et al., 2011; Wang and Dey, 2006; Xue et al., 2013; Yan et al., 2013). In the zebrafish, with a telolecithal egg, ZGA was reported to start at the 128-cell stage (8th cell cycle) (Aanes et al., 2011; Harvey et al., 2013; Mathavan et al., 2005). In other yolk-rich embryos, such as *Xenopus* and *Drosophila*, ZGA can be detected at about 128- to 256-cell (nucleus) stage (8th to 9th cell cycle) (Baroux et al., 2008; Tadros and Lipshitz, 2009). It has been hypothesized that instead of the absolute cell cycle number, the timing of ZGA in large, yolk-rich embryos is determined by the nucleocytoplasmic ratio and the maternal clock (Tadros and Lipshitz, 2009). We stained early EGK stage chick embryos with an anti-phosphorylated-RNA Polymerase II CTD (p-PolII) antibody to mark the zygotic gene transcription, DAPI to visualize the nuclei and E-cadherin to visualize cell boundaries. No specific p-PolII immunoreactive signals could be detected before late EGK-II stage (Fig. 2A, leftmost column panels). At late EGK-II/early EGK-III (Fig. 2A, 2nd and 3rd column panels), weak nuclear p-PolII signals were observed in a few centrally located cells. By mid-/late EGK-III stages (Fig. 2A, 4th and 5th column panels), p-PolII signals became progressively stronger and could be readily detected in many central cells. About half of p-PolII-positive cells had strong cytoplasmic signals at late EGK-III, a phenomenon possibly related to the cytoplasmic-to-nuclear translocation of RNA PolII at the onset of ZGA, as observed in the mouse embryo (Bellier et al., 1997). At EGK-IV (Fig. 2A, 6th and rightmost column panels; Fig. 2C,D), p-PolII signals became more robust and positive cells became more abundant. During the entire transition period from mid-EGK-II to late EGK-IV, p-PolII signals appeared in central, smaller cells sooner and more strongly than in peripheral, larger cells. However, it is unclear whether this is causally related to changes in the nucleocytoplasmic ratio because many central cells were weak or negative for p-PolII signals even when their similarly sized neighbors were strongly positive (Fig. 2C). Supporting our ZGA observation based on the p-PolII staining data, pre-ovipositional embryos with GFP transgene insertion under the control of a CMV promoter (Park and Han, 2012) showed no GFP signals at mid-EGK-II (Fig. 2B) and strong GFP signals at mid-EGK-IV (Fig. 2B). Taken together, our data suggest that ZGA in the chick is initiated at late EGK-II/early EGK-III (about 64-128 total cell stage; or approximately during the 7th or 8th cell cycle) and becomes readily detectable at mid- to late EGK-III (about 128-256 total cell-stage; 8th to 9th cell cycle). Chick embryos therefore initiate ZGA with a developmental timing comparable with that in the zebrafish and *Xenopus* embryos, pointing to potential conservation in molecular mechanisms controlling ZGA in yolk-rich embryos.

### Separation of surface and inner cells is not caused by oriented cell divisions

**Fig. 3.**
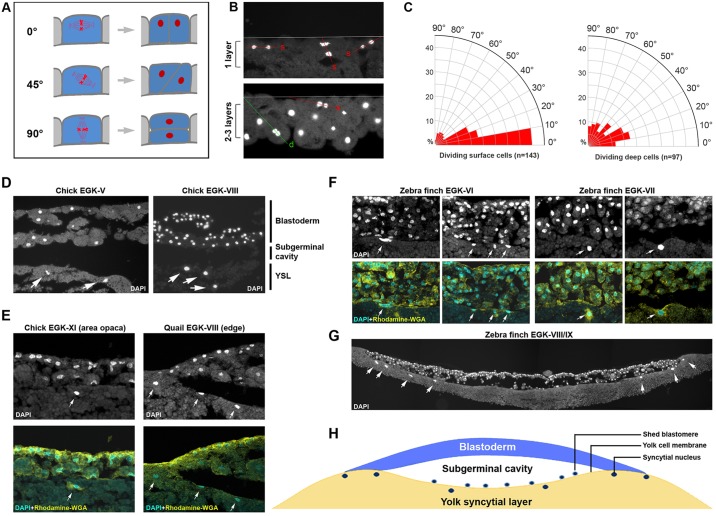
**Mitotic division orientation and yolk syncytial nuclei.** (A-C) Increase in cell layer number is not caused by oriented mitotic division. (A) Schematic view of three representative mitotic plane angles (0°, 45° and 90°). The last (90°) scenario was hypothesized as the cause for blastoderm cell-layer number increase by Eyal-Giladi and colleagues. (B) Representative section views of EGK-III to EGK-V embryos stained with DAPI to reveal mitotic cells. Top: one-cell layer region; bottom: 2- to 3-cell-layer region. Only anaphase and telophase nuclei were counted. Mitotic plane orientation was calculated as the angle between the blastoderm surface and the line passing through two separating nuclei. Red lines: surface-located cell divisions (s). Green line: deep cell division (d). (C) Rose diagrams showing the distribution of mitotic plane orientation. Left: dividing surface cells (*n*=143). Right: dividing deep cells (*n*=97). A vast majority of surface cells divide with their mitotic planes orientated at a less than 30° angle to the surface, likely producing two surface daughter cells. Mitotic planes of non-surface cells exhibit a more randomized distribution. (D-H) Yolk syncytial nuclei are detected in three different avian species. (D) DAPI staining of EGK-V (left) and EGK-VIII (right) chick embryos. DAPI-positive structures (arrows) are detected underneath the yolk cell surface. (E) In post-ovipositional chick (EGK-XI, left panels) and pre-ovipositional quail (EGK-VIII, right panels) embryos, double-staining with DAPI (nucleus) and Rhodamine-WGA (membrane) reveals that DAPI-positive signals (arrows) are located underneath the yolk cell membrane. (F) Four examples of zebra finch embryos (two EGK-VI and two EGK-VII) showing DAPI-positive syncytial nuclei (arrows) located underneath the yolk cell membrane. (G) A composite view (assembled from four images) of a zebra finch embryo cross-section, showing an intact yolk cell membrane and many syncytial nuclei (arrows) underneath it. (H) A schematic view of the YSL in an avian embryo.

From our scanning EM analysis, late EGK-II/early EGK-III is also the stage when the cellularization process becomes complete in a few centrally located cells and the increase in blastoderm cell-layer number is initiated. This latter process is significant because it results in the separation of two types of blastomeres: those located on the surface and exposed to the external environment; and those located in the inside and shielded from external influences. Eyal-Giladi and colleagues hypothesized that oriented blastomere division led to this increase (Kochav et al., 1980). In eutherian embryos, this separation is directly correlated with the future fate of these cells: trophectoderm for the surface-located cells and inner cell mass for the inside ones. As mentioned in the introduction, there are two competing, but not mutually exclusive, theories to explain this process in the mouse embryo (Johnson and Ziomek, 1981; Parfitt and Zernicka-Goetz, 2010; Sasaki, 2010; Watanabe et al., 2014). To investigate whether mitotic plane orientation is correlated with the increase in cell-layer number in the chick embryo (Fig. 3A), we performed DAPI staining with EGK-III and EGK-IV embryos in which the blastoderm was increasing its thickness from 1- to 2-cells or from 2- to 3-cells (Fig. 3B). Embryos with many mitotic cells in anaphase/telophase (e.g. as shown in supplementary material Fig. S7A,B) were selected. These embryos were sectioned and mitotic plane orientation was measured as indicated in Fig. 3B (embryo, *n*=5; mitotic nuclei pair, *n*=240). Data for dividing surface cells and dividing non-surface (deep) cells were plotted separately (Fig. 3C). The majority (about 3/4) of all surface cells divided with their mitotic plane oriented at less than 30° angle relative to the blastoderm surface, and about 40% of all surface cell divisions had an angle of less than 10° angle (Fig. 3C, left). Although dynamic cytokinetic behavior could not be visualized, those divisions most likely resulted in generating two surface daughter cells. Mitotic orientation of dividing deep cells was distributed more randomly (Fig. 3C, right), with 44% cells dividing at 0-30° angles and with the rest evenly represented at 30-90° angles. Collectively, these data indicate that cell layer number increase at cleavage stages in the chick embryo is not directly caused by oriented cell divisions.

### A yolk syncytial layer (YSL) is formed during early avian development

As mentioned in the introduction, the importance of the YSL in early development has been demonstrated in the zebrafish model. However, whether a similar YSL exists in the avian embryo has not been investigated. DAPI staining and section analysis suggested that no syncytial nuclei could be found at stages EGK-I to -IV (not shown). At EGK-V, occasional syncytial nuclei were detected (Fig. 3D, left), which became brighter, larger and more abundant at later EGK stages (Fig. 3D, right, showing an EGK-VIII embryo) and persisted through post-ovipositional stages (Fig. 3E, left, showing an EGK-XI embryo). These nuclei are to be distinguished from DAPI-positive cells located above the surface of the yolk cell (e.g. as shown in Fig. 3G; supplementary material Fig. S7F), which are shed from the blastoderm cell mass (as described by Eyal-Giladi and Kochav) and are frequently observed during the blastoderm thinning process from EGK-VII onwards. To confirm the existence of a YSL in the avian embryo, we performed similar analysis with quail (Fig. 3E, right) and zebra finch (Fig. 3F,G) embryos. Pre-ovipositional quail eggs were retrieved and DAPI staining of these embryos revealed the presence of syncytial nuclei, especially underneath the area opaca and close to the blastoderm margin (Fig. 3E, right). Zebra finch eggs are laid at an earlier developmental stage than chicken or quail eggs, and zebra finch embryos at stages EGK-VI to -IX can be collected easily from freshly laid eggs (S. S. Mak, C. Alev, H.N., A. Wrabel, Y. Matsuoka, A. Honda, G. Sheng and R. K. Ladher, unpublished). DAPI staining and section analysis of EGK-VI to -IX zebra finch embryos supported our chick and quail data (Fig. 3F,G). Syncytial nuclei were detected more abundantly in the zebra finch (Fig. 3G, arrows) and co-staining with a membrane marker (rhodamine-labeled WGA) revealed that these DAPI-positive signals were located underneath the yolk cell membrane (Fig. 3F). Interestingly, many peripherally located syncytial nuclei in the finch embryo could be found even in whole-mount views (supplementary material Fig. S7C-E). There nuclei were located away from the blastoderm edge, as revealed by phalloidin co-staining (supplementary material Fig. S7D,D′,E,E′) and in section (supplementary material Fig. S7F). Taken together, these data strongly support the hypothesis (Fig. 3H) that a YSL is present in the avian embryo. Whether this YSL plays a role in patterning the overlying blastoderm remains to be clarified.

### Summary

The first half of pre-ovipositional chick development, from EGK-I to EGK-V, was investigated in this study (Table 1). During this developmental period, a chick embryo undergoes ∼11 rounds of mitotic divisions. The cell number increases from one to ∼2000 and the cell-layer number increases from one to four. Meroblastic cleavages continue from EGK-I, when all cells are open, to late EGK-V, when all cells are fully cellularized. Blastomere-yolk separation starts at late EGK-II and ZGA starts at late EGK-II/early EGK-III (∼7th to 8th nuclear division cycle). Separation of outside and inside blastomeres is not due to oriented cell division, as previously suggested. Syncytial nuclei located underneath the yolk cell membrane can be detected from EGK-V and become more abundant during the second half of pre-ovipositional development. The presence of yolk syncytial nuclei is also observed in quail and zebra finch embryos. Our data on blastoderm cell-layer increase support the hypothesis that this process is controlled by cellular mechanisms other than oriented mitotic division. Collectively, our data suggest that many features of cleavage-stage development in the chick resemble those in the zebrafish. Whether this conservation is a result of convergent evolution or is indicative of hardwired molecular and cellular mechanisms regulating vertebrate early development awaits future investigation.

**Table 1.**
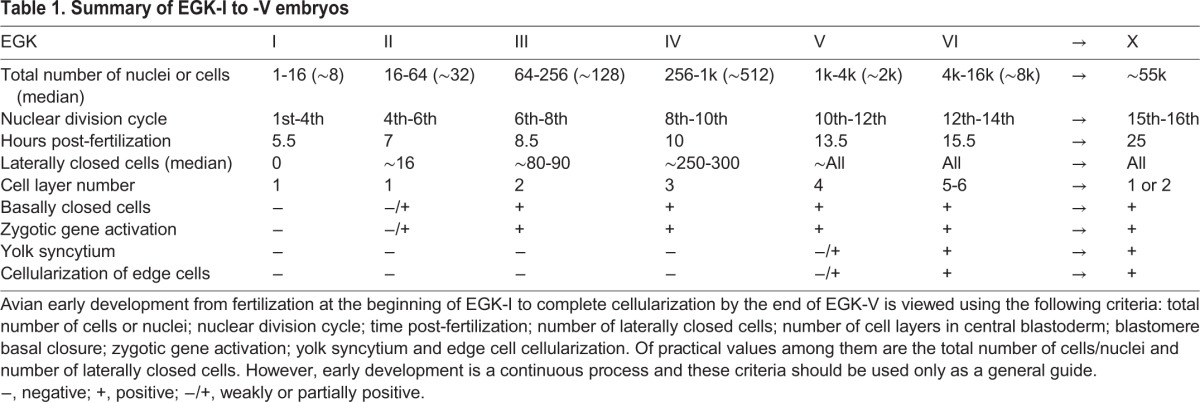
**Summary of EGK-I to -V embryos**

## MATERIALS AND METHODS

### Embryo collection

EGK stage chick embryos were collected from laying white leghorn hens using a non-invasive, manual retrieval (abdominal massage) method as previously described (Lee et al., 2013a). The retrieval was carried out in the afternoon or early evening and was timed to target eggs in the first half of pre-ovipositional development. Each collected egg was placed in a Petri dish. The eggshell was opened and removed, and the egg yolk was adjusted so that the embryo was positioned at the top. Excessive albumen covering the embryo was removed and a few drops of ice-cold 4% paraformaldehyde (PFA) fixative (Alev et al., 2013) were put on the vitelline membrane over the embryo to preserve the morphology. After a few minutes of fixation at room temperature, the embryo, together with a small amount of peripheral and underlying yolk, was excised from the bulk of the yolk and placed in Pannett-Compton saline solution (Alev et al., 2013). The vitelline membrane was then carefully removed and the embryo together with attached yolk was transferred to 4% PFA for further fixation and storage at 4°C. Out of 138 embryos obtained this way, 85 embryos were analyzed for stage distribution under a stereo microscope, and 73 out of 85 were within the cleavage stages according to the EGK staging system (13 EGK-I, 16 EGK-II, 15 EGK-III, 17 EGK-IV and 12 EGK-V). Pre-ovipositional GFP transgenic chick embryos and pre-ovipositional quail embryos were collected using the same abdominal massage method. Post-ovipositional zebra finch eggs were collected daily from locally maintained colonies and zebra finch embryos were processed for staining using the same protocol as for the chick embryos.

### Scanning electron microscopy (SEM)

Samples for SEM analysis were prepared as described previously (Ikenouchi et al., 2013). Briefly, EGK stage chick embryos were fixed with 2.5% glutaraldehyde and 2% formaldehyde in 0.1 M sodium cacodylate buffer (pH 7.4) for 2 h at room temperature, followed by post-fixation with 1% OsO_4_ in the same buffer for 2 h on ice. Post-fixed embryos were dehydrated in ethanol and then transferred to isoamyl acetate, followed by critical point drying (JCPD-5, JEOL). Specimen surface was coated using an osmium coater (Neoc-STB, Meiwafosis). For fracture surface images after whole-mount SEM, embryo specimen was settled on a small piece of adhesive tape, and fractured using a fine tungsten needle. All SEM images were acquired using scanning electron microscope JSM-5600LV (JEOL) with accelerating voltage set at 10 kV.

### Immunofluorescence, nuclear staining, imaging and image analysis

All steps except for the imaging were carried out at 4°C. For phosphorylated RNA polymerase II detection, fixed embryos were washed in PBTw (PBS with 0.1% Tween-20) for 3×20 min, followed by permeabilization in PBTr (PBS with 0.1% Triton X-100) for 3×30 min and by blocking in blocking solution (PBS with 1% DMSO, 0.1% Triton X-100 and 5% skimmed milk). Blocked embryos were incubated overnight with anti-RNA polymerase II CTD pSer5 (1:1000, Abcam Cat# ab5131), washed in PBTw, reblocked in blocking solution and incubated in the secondary antibody (Alexa Fluor 594 goat anti-rabbit IgG(H+L) (1:300, Molecular Probes Cat# a11012) before imaging. DAPI (Molecular Probes, D1306) and 36/E-Cadherin antibody (BD Transduction Laboratories, 610182) were used for whole-mount nuclear and E-cadherin staining, respectively. Yolk cell membrane was marked using rhodamine-labeled wheat germ agglutinin (WGA) (Vector Laboratories, Cat# RL-1022). Whole-mount fluorescence images were acquired using Olympus FV1000 microscope. Section fluorescence images were acquired using Olympus BX51W1 or Olympus FV1000 microscope after processing for 10 µm paraffin-embedded section or 12 µm cryosection followed by mounting in ProLong Gold antifade reagent with DAPI (Molecular Probes Cat# P36931). Mitotic division angles were measured using Fiji software and data were plotted as rose diagrams using GeoRose software.

## Supplementary Material

Supplementary Material
