# Supplementary material for: Cellular analysis of cleavage-stage chick embryos reveals hidden conservation in vertebrate early development
Source: Development. 2015 Apr 1;142(7):1279–86. doi: 10.1242/dev.118604 (PMC4378249; doi:10.1242/dev.118604)
Supplement: Supplementary Material [file supp_142_7_1279__index.html]

Supplementary Material 

# Cellular analysis of cleavage-stage chick embryos reveals hidden conservation in vertebrate early development

## DEV118604 Supplementary Material

**Files in this Data Supplement:**

- Supplementary Material
