# Supplementary material for: Cellular analysis of cleavage-stage chick embryos reveals hidden conservation in vertebrate early development
Source: Development. 2015 Apr 1;142(7):1279–86. doi: 10.1242/dev.118604 (PMC4378249; doi:10.1242/dev.118604)
Supplement: Supplementary Material [file supp_dev.118604_DEV118604supp.pdf]

Supplementary figures

Figure S1 Surface view of EGK-I to -V embryos under a stereomicroscope
For each EGK stage, top panel shows whole-embryo view and bottom panel shows magnified view of the blastoderm center. Scale bar (same in all whole-embryo panels): 1 mm. For EGK-I, -II and –III, two embryos of different sub-stages are shown.

Figure S2 SEM of EGK-I embryo
A) Whole-embryo view. **B)** Magnified view of A, with each cell labeled by a number. Arrowheads: fracture positions and view directions in C and D. Line: fracture position in G. **C,D)** Fracture surface views showing incomplete cellularization and transition in cleavage furrow from vertical to horizontal burrowing. Arrowheads: areas with magnified views shown in E and F. **E)** View from outside the plasma membrane, showing elaborate membrane protrusions at the base of cell No.6. The basal membrane separating cell No.6 and the yolk structure (yolk cell) is also visible. **F)** Magnified view of the apical surface of cell No.5. Microvilli are abundant and many vesicular structures are present at the cortex. **G)** Yolk granules are distributed throughout the cytoplasm. Their sizes decrease gradually as they approach the surface. No morphological boundary can be identified between those to be allocated to the future blastomeres and those to the yolk cell. Scale bars: as indicated.

Figure S3 SEM of EGK-II embryo
A) Whole-embryo view. **B)** Magnified view of A, with numbers indicating cells at the fracture surface shown in C. **C)** Fracture surface view, showing central cells with their basal side already separated from the yolk cell. **D)** Magnified view of C. Numerous vesicular structures are present in both cellularized (D) and open cells (E). Scale bars: as indicated.

Figure S4 SEM of EGK-III embryo
A) Whole-embryo view. **B)** Magnified view of A. Arrowheads in A: areas with higher magnification shown in C and D. **C)** Peripheral cells are still open. **D)** Numerous microvilli are present at the apical surface, being less abundant at the cleavage furrow. **E)** Subgermical cavity starts to form locally. **F)** The blastoderm is 2-cell thick in some areas. Scale bars: as indicated.

Figure S5 SEM of EGK-IV embryo
A) Whole-embryo view. **B)** Magnified view of A. The apical surface of most cells is not flattened yet. **C-E)** Appearance of a subgerminal cavity (sc; arrowheads) is widely seen under most central cells. The cavities start to merge as a continuous, but irregular shaped space. The blastoderm has 3-cell layers in its thickest part (E), decreasing to 2-cell (D) and 1-cell (C) in the peripheral regions. **F)** A peripheral edge cell in the process of cellularization. The basal membrane of this cell has formed already, whereas its peripheral side is still connected to the yolk. **G,G')** Trapped sperm (arrowheads) are occasionally visible. **H,H')** Some peripheral cells have just completed the cellularization process, with rudimentary subgerminal cavity (sc) as a small pocket between two blastomeres (No.1 and No.2) and the yolk cell. **H')** Underneath newly formed blastomere (No.2), two plasma membranes (one of blastomere and the other of the yolk cell; shown by arrows) can be clearly distinguished. No subgerminal cavity has formed there yet. An abrupt shift in yolk granule sizes (arrowheads) is often seen between the yolk cell and the newly formed blastomere. Scale bars: as indicated.

Figure S6 SEM of EGK-V embryo
A) Whole-embryo view. **B)** Magnified view of A. **C,D)** Fracture surface views from the most peripheral (leftmost cell in C) to the blastoderm center (rightmost in D). Subgerminal cavity (sc) is now an expanded, continuous space. The blastoderm is 1-cell thick at the periphery and 4-cell thick in the center. Many, but not all, peripheral edge cells have now completed the cellularization process (example shown in **C**,**E**). Blastomeres in the 1-cell thick region maintain tight association with the yolk cell membrane. In transition zone from 1- to 2-cell thick area, wedge-shaped blastomeres are often observed (**C**,**F**). Blastomeres in multilayered regions have abundant small protrusions and maintain tight association to each other (**F,G,H,I**). The apical surface now has much more flattened morphology (**B,J**) than at previous stages. Scale bars: as indicated.

Figure S7 Examples of chick embryo with many mitotic cells and of zebra finch embryos with many syncytial nuclei
A,A') A DAPI-stained EGK-III chick embryo. **B,B')** A DAPI-stained EGK-IV chick embryo. **A'**: magnified view of central cells in A. **B'**: magnified view of peripheral cells in B. Peripheral cells often exhibit synchrony in mitotic divisions (B'), whereas central cells divide asynchronously (A'). **C)** whole-mount, apical surface view of a DAPI-stained EGK-VIII finch embryo. Many positive signals are detected peripheral to the blastoderm edge. **D,D',E,E')** A finch embryo co-stained with phalloidin (D,E) and DAPI (D',E'). E and E' are magnified views of D and D', respectively. Blastoderm edge is clearly visible in D and E. **F)** Section view of an EGK-VIII finch embryo, showing a syncytial nucleus (arrowhead) and many shed blastomeres located above the yolk cell membrane (stippled line). **G)** Section view of an EGK-VIII finch embryo, showing a syncytial nucleus located peripheral to the blastoderm edge.

Figure S1

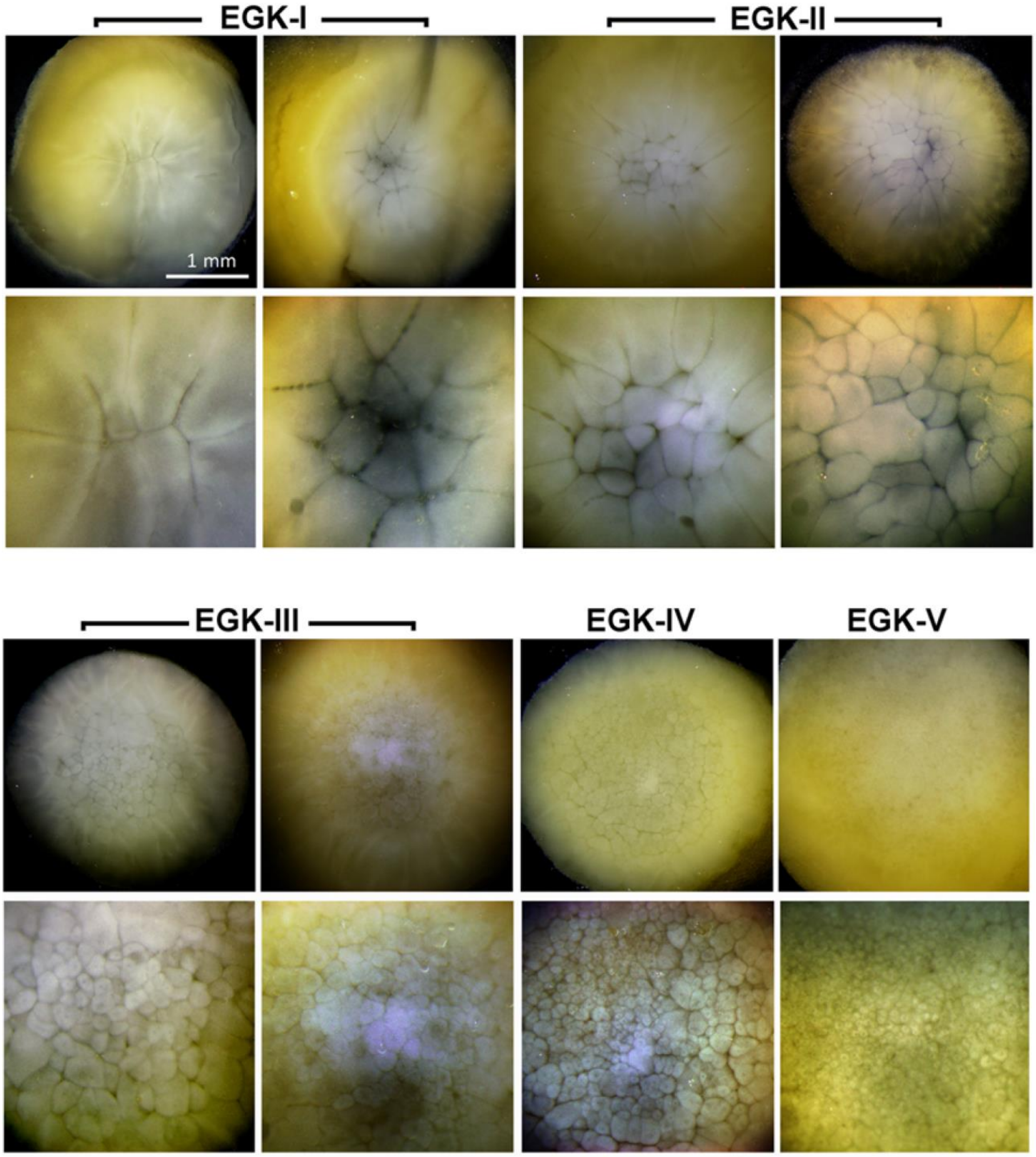

Figure S2

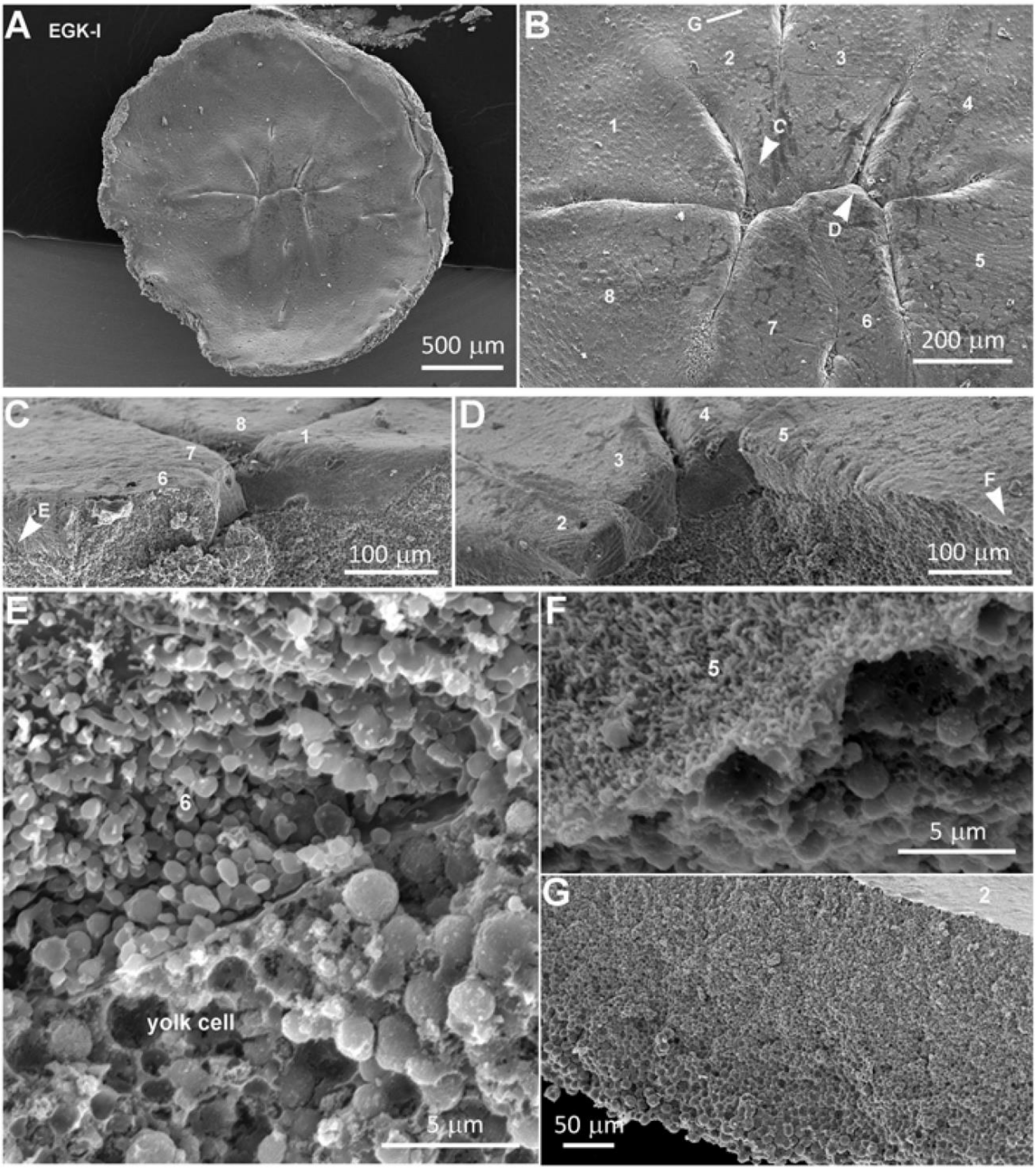
A
EGK-I
500 µm
B
G
1
2
3
4
5
6
7
8
C
D
200 µm
C
8
1
7
6
E
100 µm
D
4
5
3
2
F
100 µm
E
6
yolk cell
5 µm
F
5
5 µm
G
2
50 µm

Figure S3

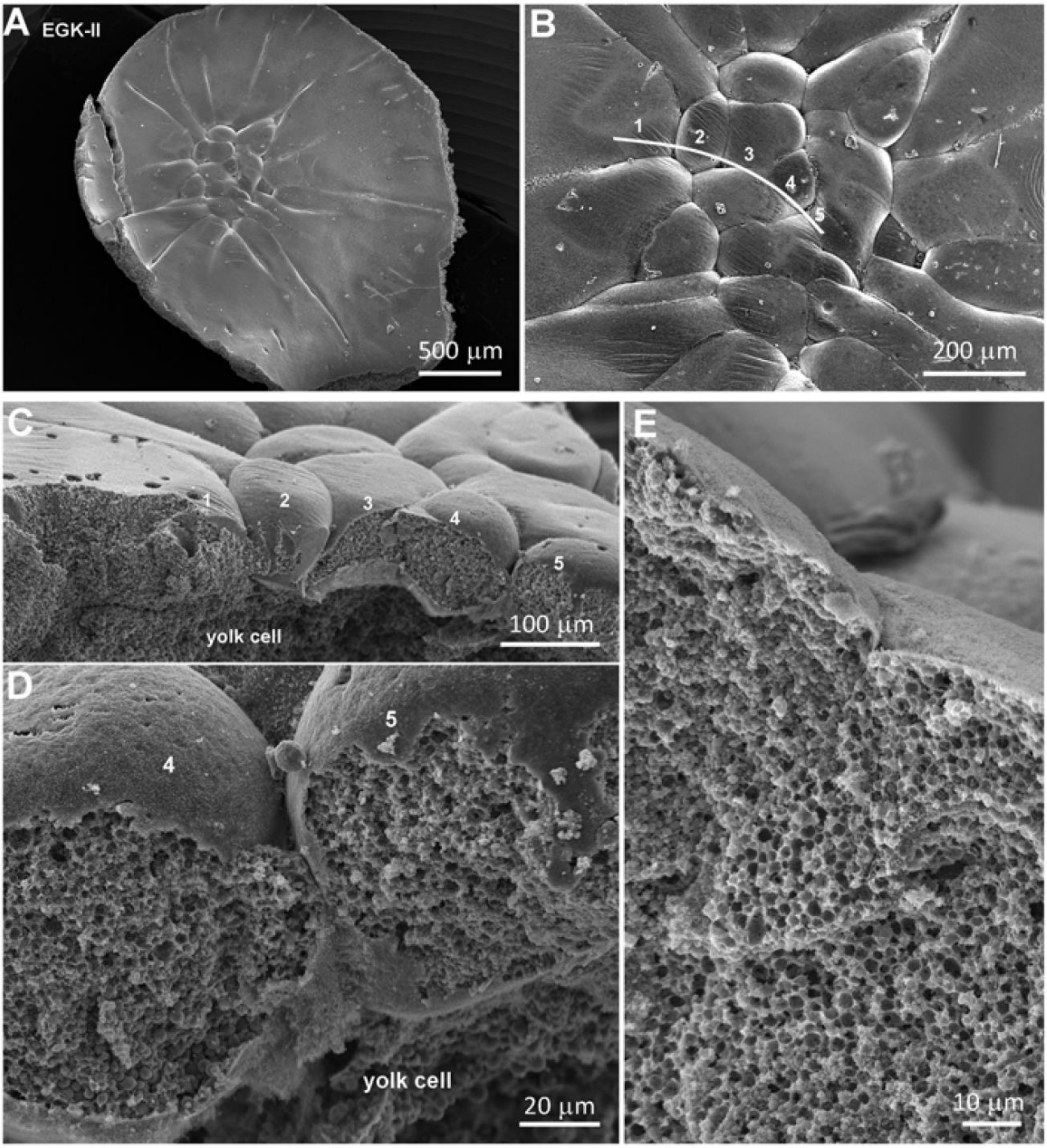
A
EGK-II
500 µm
B
1
2
3
4
5
200 µm
C
1
2
3
4
5
yolk cell
100 µm
D
4
5
yolk cell
20 µm
E
10 µm

Figure S4

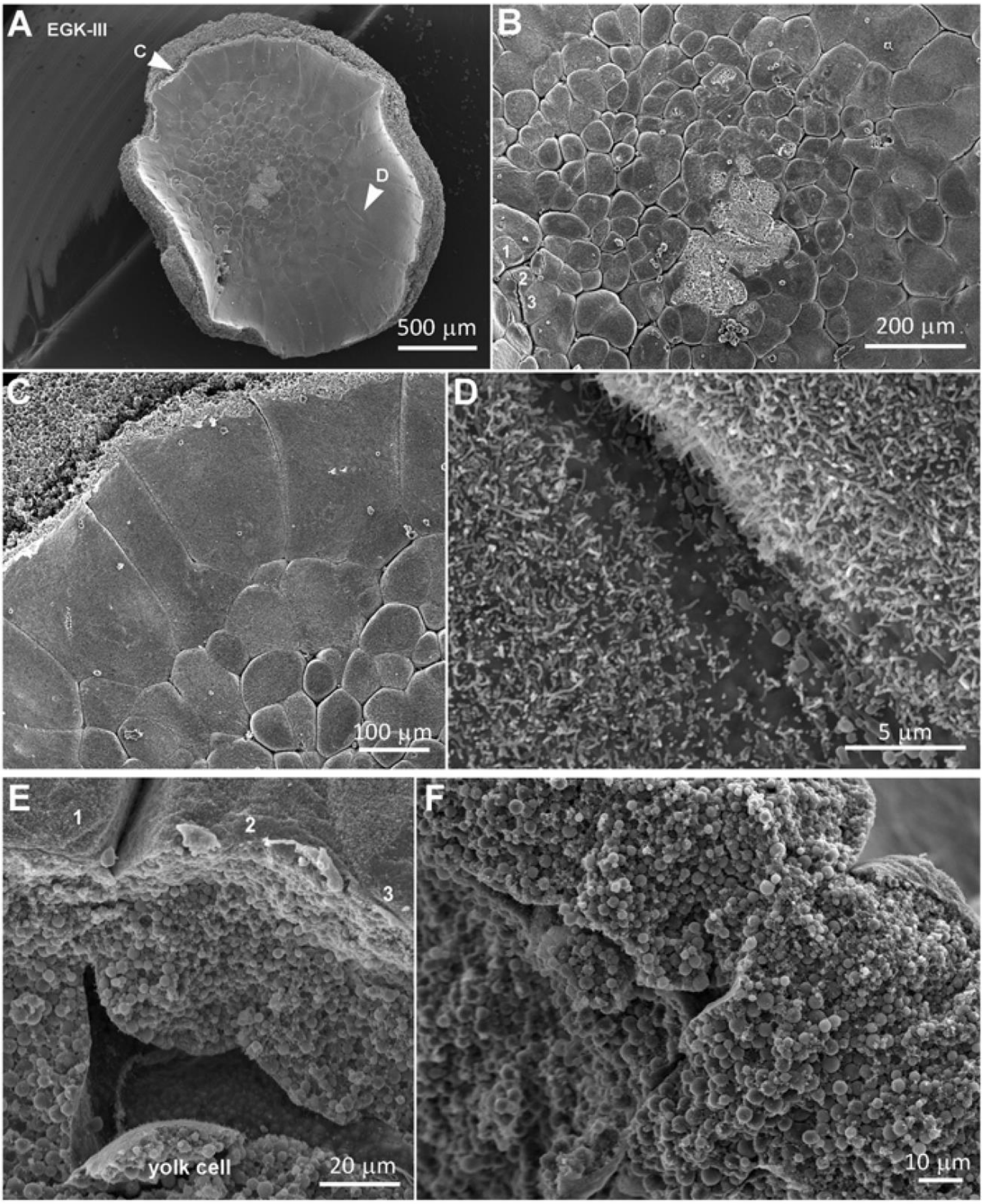
A
EGK-III
C
D
500 µm
B
1
2
3
200 µm
C
100 µm
D
5 µm
E
1
2
3
yolk cell
20 µm
F
10 µm

Figure S5

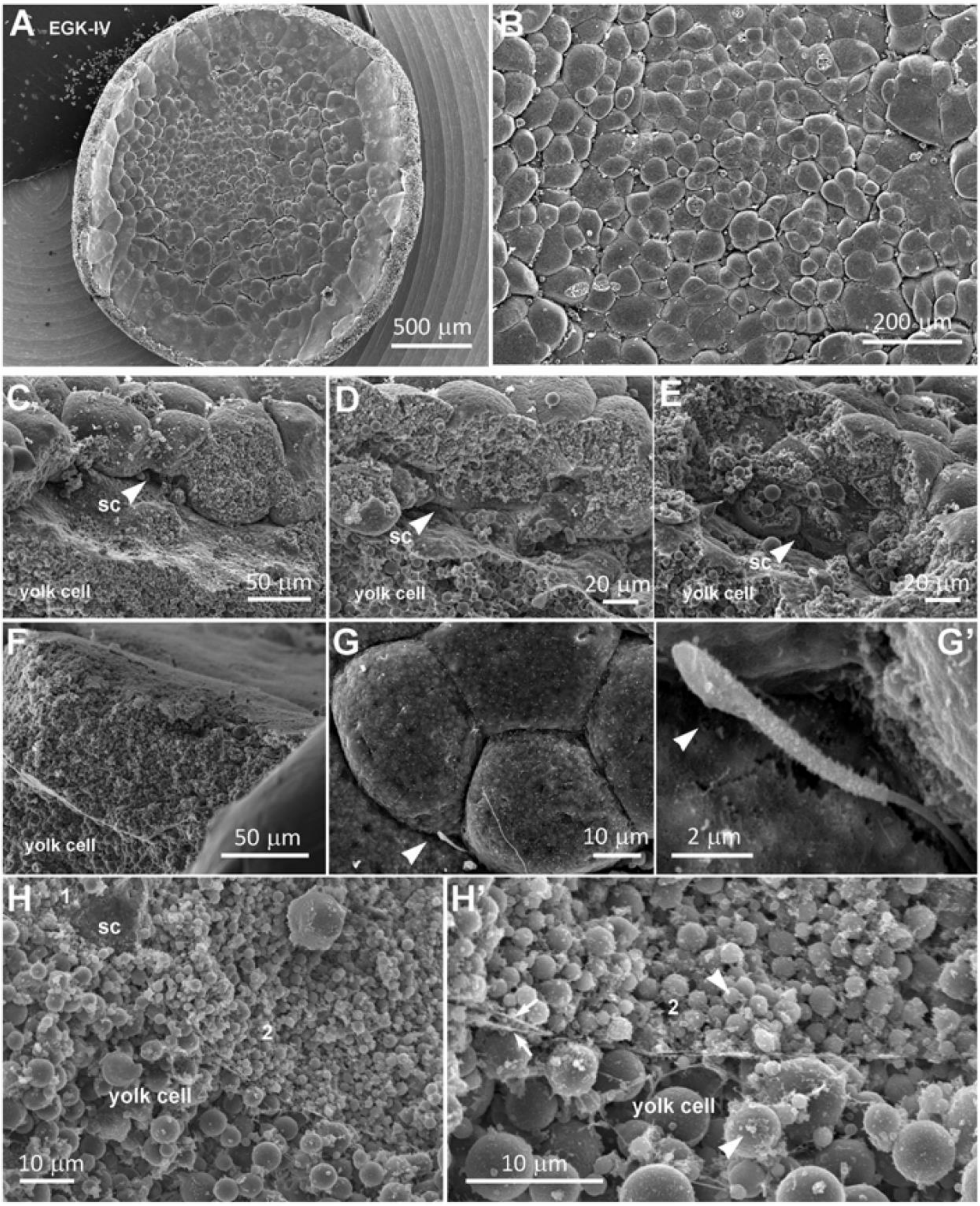
A
EGK-IV
500 µm
B
200 µm
C
sc
yolk cell
50 µm
D
sc
yolk cell
20 µm
E
sc
yolk cell
20 µm
F
yolk cell
50 µm
G
10 µm
G'
2 µm
H
1
sc
2
yolk cell
10 µm
H'
2
yolk cell
10 µm

Figure S6

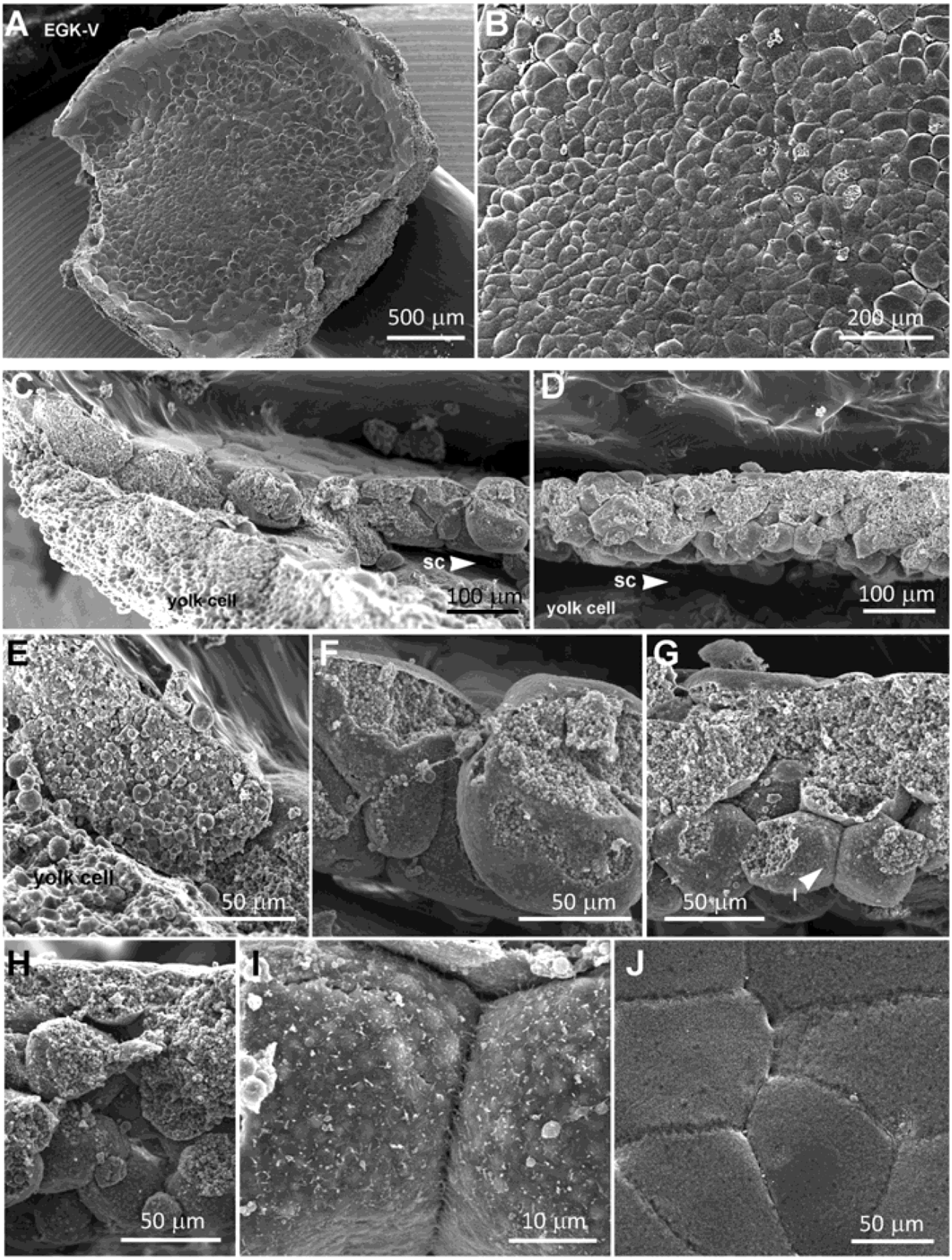
A
EGK-V
500 µm
B
200 µm
C
sc
yolk cell
100 µm
D
sc
yolk cell
100 µm
E
yolk cell
50 µm
F
50 µm
G
50 µm
H
50 µm
I
10 µm
J
50 µm

Figure S7

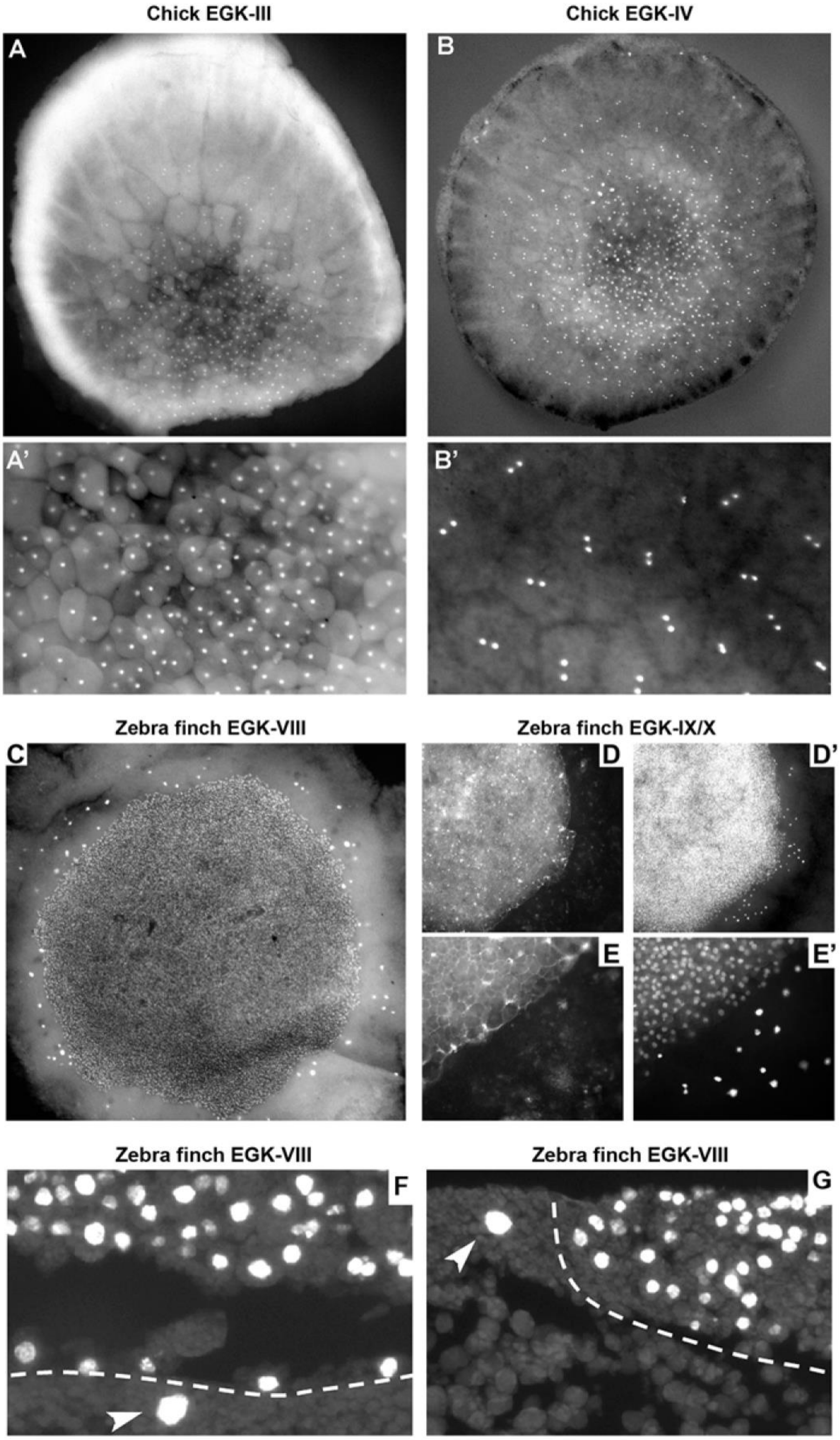
Chick EGK-III
Chick EGK-IV
A
B
A'
B'
Zebra finch EGK-VIII
Zebra finch EGK-IX/X
C
D
D'
E
E'
Zebra finch EGK-VIII
Zebra finch EGK-VIII
F
G
